# Blood product transfusions in septic patients are associated with mortality, ARDS, and more days on mechanical ventilation

**DOI:** 10.1186/cc13301

**Published:** 2014-03-17

**Authors:** PP Dobesh, DG Klepser, TR McGuire, DA Roberts, AL Himmelberg, KM Olsen

**Affiliations:** 1 University of Nebraska Medical Center College of Pharmacy, Omaha, NE, USA

## Introduction

The objective of this study was to determine whether septic patients requiring blood product transfusions have worse outcomes compared with those who do not require transfusions. It is not uncommon for patients with sepsis to require blood product transfusions. The need for transfusions may be indicative of inflammatory consumption of blood cells, active blood loss, or impaired hematopoiesis. Regardless of the etiology, need for transfusion may be an indicator of a more severely ill patient and a valuable prognostic factor.

## Methods

This retrospective cohort study included all patients over the age of 40 years with a confirmed diagnosis of sepsis and an ICU stay at our academic medical center from 1 January 2005 to 31 March 2011. Use of blood product transfusion, patient demographics, and APACHE II score at the time of sepsis were collected from patient charts. Outcomes of interest were in-hospital mortality, development of acute respiratory distress syndrome (ARDS), days on mechanical ventilation, hospital cost, and length of stay.

## Results

We identified 824 patients who met the inclusion criteria for this study. Of those patients, 543 (66%) received at least 1 unit of blood products during hospitalization. Patients receiving blood products had significantly higher in-hospital mortality (36.1% vs. 26.9%; *P *= 0.003) and a higher rate of development of ARDS (45.3% vs. 27.1%; *P *< 0.001) compared with patients not receiving blood products. Patients receiving packed red blood cells (PRBCs) (60%) did not demonstrate a significant increase in mortality (35.1% vs. 28.8%; *P *= 0.058), while patients receiving platelets (20%) did have higher mortality (45.1% vs. 29.5%; *P *< 0.001). Transfusions of PRBCs or platelets were both associated with a higher development of ARDS (*P *< 0.001 for both). There was a significant increase in days on mechanical ventilation (7.0 days vs. 2.3 days; *P *< 0.001), hospital cost ($88,331 vs. $35,047; *P *< 0.001), and length of stay (17.3 days vs. 9.7 days; *P *< 0.001) for patients receiving blood products, regardless of the type. These differences were seen despite the mean APACHE II score being similar (22.8 vs. 22.5; *P *= 0.645).

## Conclusion

Patients with sepsis receiving blood products, particularly platelets, were significantly more likely to develop ARDS, had more days on mechanical ventilation, and had higher mortality. The lack of an increase in mortality associated with PRBC transfusion may be due to the benefit in oxygen delivery or sample size.

